# Sex differences in severe acute malnutrition in children under 5 years in Zambia

**DOI:** 10.1371/journal.pgph.0004707

**Published:** 2025-06-23

**Authors:** Gift C. Chama, Situmbeko Liweleya, Lweendo Muchaili, Bislom C. Mweene, Sydney Mulamfu, Lukundo Siame, Sepiso K. Masenga

**Affiliations:** 1 Department of Physiological Sciences, Mulungushi University, School of Medicine and Health Sciences, Livingstone, Zambia; 2 Department of Cardiovascular Science and Metabolic Diseases, Livingstone Center for Prevention and Translational Science, Livingstone, Zambia; Islamic Azad University South Tehran Branch, IRAN, ISLAMIC REPUBLIC OF

## Abstract

Severe acute malnutrition (SAM) is a critical public health issue, particularly in low and middle-income countries like Zambia, where it significantly contributes to under-five mortality. While general factors associated with SAM, including its overall burden, are well documented, the role of sex differences in correlates of SAM has not been thoroughly explored. Hence, this study aimed to examine sex differences in correlates of SAM by identifying key factors influencing malnutrition in males and females under five years, with particular attention to comorbid conditions such as Human Immunodeficiency Virus (HIV) and Tuberculosis (TB), which are known to complicate malnutrition in this population. We conducted a retrospective cross-sectional study utilizing data from 429 medical records of children aged 6 months to 5 years who were attended to at Livingstone University Teaching Hospital between 2020 and 2022. The median age at diagnosis for both males and females was 18 months, with interquartile ranges of 11–25 months and 12–24 months, respectively. Females had a higher prevalence of SAM (24.3%, n = 46) compared to males (19.58%, n = 47). TB was significantly associated with SAM in both males (AOR: 14.30, 95% CI: 2.08–98.5, p = 0.006) and females (AOR: 40.50, 95% CI: 4.83–340, p < 0.001), and the lymphocyte-to-monocyte ratio was also associated with SAM in males (AOR: 1.39, 95% CI: 1.05–1.83, p = 0.017) and females (AOR: 1.22, 95% CI: 1.00–1.49, p = 0.045). Additionally, comorbidities (AOR: 4.1, 95% CI: 1.13–14.90, p = 0.031) and age (AOR: 0.91, 95% CI: 0.85–0.97, p = 0.009) were associated with SAM in females, while these associations were not significant in males. Overall, females are more frequently diagnosed with SAM, most likely due to the presence of comorbidities such as TB and HIV. TB was found to be a critical risk factor for SAM in both sexes, highlighting the need for sex-specific interventions in the management of SAM.

## Introduction

Severe acute malnutrition (SAM) is one of the leading causes of morbidity and mortality in children under five years globally, particularly in low–income regions like Sub-Saharan Africa, where poverty, food insecurity, and limited healthcare access prevail [1–3]. Our previous study carried out in Zambia underscores this concern, revealing an alarming SAM prevalence of 27%, which further supports the assertion that SAM is a critical issue in Sub-Saharan Africa [4]. However, the role of sex differences in undernutrition has not been extensively explored in this region.

While undernutrition affects all children, there is increasing evidence that sex differences may influence both the prevalence and outcomes of SAM, more especially in low-and middle-income countries with a positive correlation between child mortality and morbidity with the prevalence of SAM [5,6]. Some studies have shown that males are more susceptible to undernutrition in comparison to females, and they are likely to be wasted, stunted or underweight [7,6], while other studies found that females were more vulnerable to undernutrition due to sociocultural factors such as food distributions biases and care-seeking behaviors [8–10]. Other reasons attributed include, biological factors, including immune and endocrine system variations, may also contribute to these disparities [9–11]. Studies have also shown that malnourishment in children alters hematological parameters in comparison to children who are not malnourished [12], but the sex differences are usually not reported.

Understanding sex-specific differences in correlates of SAM is crucial for designing targeted interventions to improve treatment outcomes and the underlying reasons for these differences remain poorly understood, as studies focusing on sex differences are limited. Hence, this study aimed to analyze sex differences in the prevalence of SAM in children under five years, as well as identify key risk factors contributing to these differences, and explore hematological factors including key comorbid conditions such as HIV and TB.

## Methods

### Study design and site

This was a retrospective chart review conducted between 14 august 2023 and 8 January 2024. We utilized secondary data from a previous study conducted at Livingstone University Hospital (LUTH) among children under 5 years with and without SAM who attended the hospital between 2020–2022 [4].

### Eligibility and recruitment

**Fig 1:**
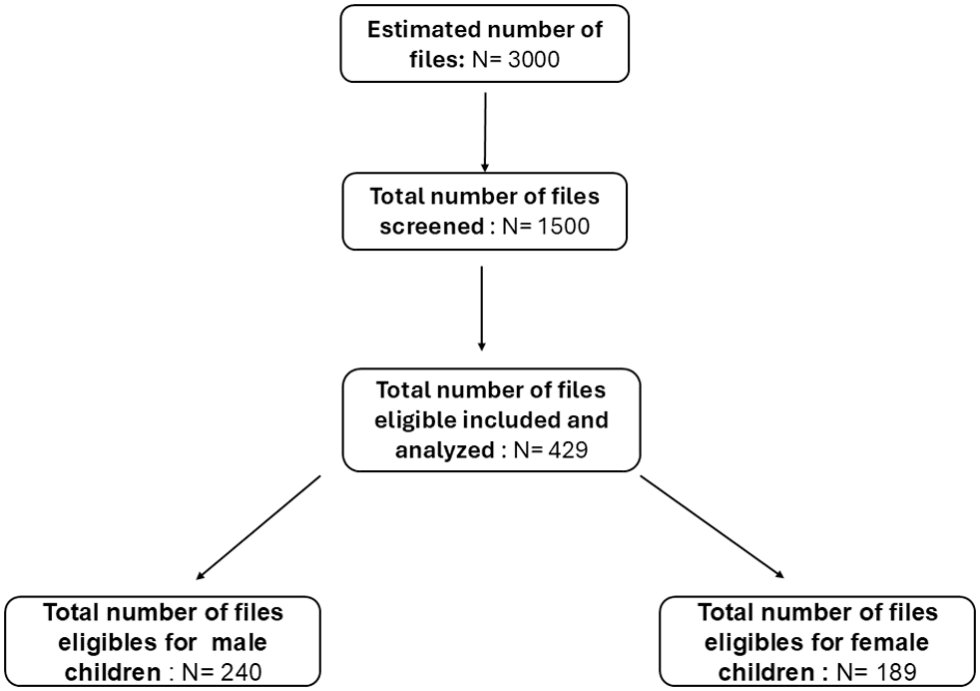
Flow diagram of screened and eligible flies.

The eligibility criteria, recruitment, study procedures and demographic characteristics of this population have been described in detail in our previous study [4]. Out of a total of 1,500 files screened, 429 files of children aged 6–59 months were found eligible. Of these, 240 files were for male children and 189 for female children, all of which were included in the analysis. A total of 1071 files were excluded as they were incomplete and were missing vital information such as age, sex and malnutrition status (Fig 1).

### Study variables

The primary outcome variable in this study was SAM and it was diagnosed based on our previous study [4]. Briefly, the diagnosis of SAM was based on the World Health Organization (WHO) criteria, including a weight-for-height (WFH) Z-score below -3 standard deviation, a mid-upper arm circumference (MUAC) less than 115 mm, or the presence of bilateral pitting edema. The independent variables encompassed demographic (age and residence), clinical HIV status, type of TB (bacteriologically or clinically confirmed), comorbidities (sickle cell disease, anemia, pneumonia, acute diarrhea disease, cerebral palsy, acute kidney injury, congenital heart disease, asthma, diabetes mellitus and lymphoma) and hematologic parameters. Because HIV and TB are the most reported comorbidities, they were treated as distinct categories, rather than grouped with other comorbidities.

### Data analysis

Data were analyzed using Stat Crunch software. Descriptive statistics such as median, standard deviation and interquartile range were used to summarize the characteristics of the study population while logistic regression was implored to identify independent predictors and adjust for confounders.The variables selected were based on prior literature and those that were significant in univariable analysis [13,14].

### Ethical approval and consent to participant

Ethics approval for this study was obtained from the Mulungushi University School of Medicine and Health Sciences (SOHMS) Research Ethics Committee (ethics reference number SMHS-MU2-2023-64) on 6^th^ June 2023. To guarantee complete confidentiality and anonymity, all data collected was de-identified. Written or verbal consent was not applicable and therefore waived by the ethics committee as secondary data were used in this study.

To strengthen the reporting for this observational study, we made use of the Strengthening the Reporting of Observational Studies in Epidemiology (STROBE), S1 Checklist.

## Results

### Comparison of sociodemographic and clinical factors between males and females with and without severe acute malnutrition in the whole population

The study involved 429 participants, with males comprising 55.9% (n = 240) and SAM being more prevalent in females (24.3%, n = 46) (Table 1**).** The median age of males at the time of diagnosis was 18 months (interquartile range (IQR) 11–25), compared to 18 months (IQR 12–24) for females. The majority were from urban areas, with males comprising 62.5% (n = 150) and females 64% (n = 121) (Table 1). Overall, HIV and TB were more prevalent in males, but both were associated with SAM in males and females while comorbidities were only associated with SAM in females and not males (Table 1).

**Table 1:** Socio-demographic characteristics by sex differences.

	Males				Females			
Variables	Frequency (%)	SAM		P-value	Frequency (%)	SAM		P-value
		Yes=19.58% (n=47)	No = 80.41% (n=193)			Yes= 24.3% (n=46)	No=75.66% (n=143)	
**Age, months**, *median (IQR*)	18 (11, 25)	17(12, 24)	18 (11, 29)	0.572	18 (12, 24)	16.9 (14,24)	19 (12,36)	0.494
**Residence**								
Urban	150 (62.50)	26 (17.33)	124 (82.67)	0.256	121 (64.0)	27 (22.31)	94 (77.69)	0.386
Rural	90 (37.50)	21 (23.33)	69 (76.67)		68 (36.0)	19 (27.94)	49 (72.06)	
**HIV Status**								
With HIV	27 (11.60)	17 (62.96)	10 (37.04)	<0.001	15 (8.3)	7 (46.67)	8 (53.33)	0.035
Without HIV	206 (88.40)	29 (14.08)	177 (85.92)		166 (91.7)	37 (22.29)	129 (77.71)	
**Tuberculosis**								
Yes	25 (10.40)	17 (68.00)	8 (32.00)	<0.001	19 (10.10)	16 (84.21)	3 (15.79)	<0.001
No	215 (89.60)	30 (13.95)	185 (86.05)		170 (89.90)	30 (17.65)	140 (82.35)	
**Type of tuberculosis**								
Pulmonary	13 (52.00)	8 (61.54)	5 (38.46)	0.672	11 (57.90)	9 (81.82)	2 (18.18)	1.000
Extra pulmonary	12 (48.00)	9 (75.00)	3 (25.00)		8 (42.10)	7 (87.50)	1 (12.50)	
**Comorbidities**				0.050				< 0.001
Yes	54 (22.50)	16(29.63)	38 (70.37)		48 (25.40)	21 (43.75)	27 (56.25)	
No	186 (77.50)	31 (16.67)	155 (83.33)		141 (74.60)	25 (17.73)	116 (82.27)	
**Top Comorbidities**				0.560				0.132
Sickle cell disease	3(9.4)	0(0.00)	3 (12.50)		10 (50.0)	1 (25.00)	9 (56.25)	
Acute watery diarrhea	8 (25.00)	3 (37.5)	5 (20.83)		4 (20.00)	0 (0.00)	4 (25.00)	
Anemia	8 (25.00)	3 (37.50)	5 (20.83)		2 (10.00)	1 (25.00)	1 (6.25)	
Pneumonia	6 (18.75)	1 (12.50)	5 (20.83)		3 (15.00)	2 (50.00)	1 (6.25)	
Acute pharyngotonsillitis	7 (21.88)	1 (12.50)	6 (25.00)		1 (5.00)	0 (0.00)	1 (6.25)	

IQR, Interquartile range, HIV, Human Immunodeficiency Virus, comorbidities (sickle cell disease, anemia, pneumonia, acute diarrhea disease, cerebral palsy, acute kidney injury, congenital heart disease, asthma, diabetes mellitus and lymphoma).

**Fig 2:**
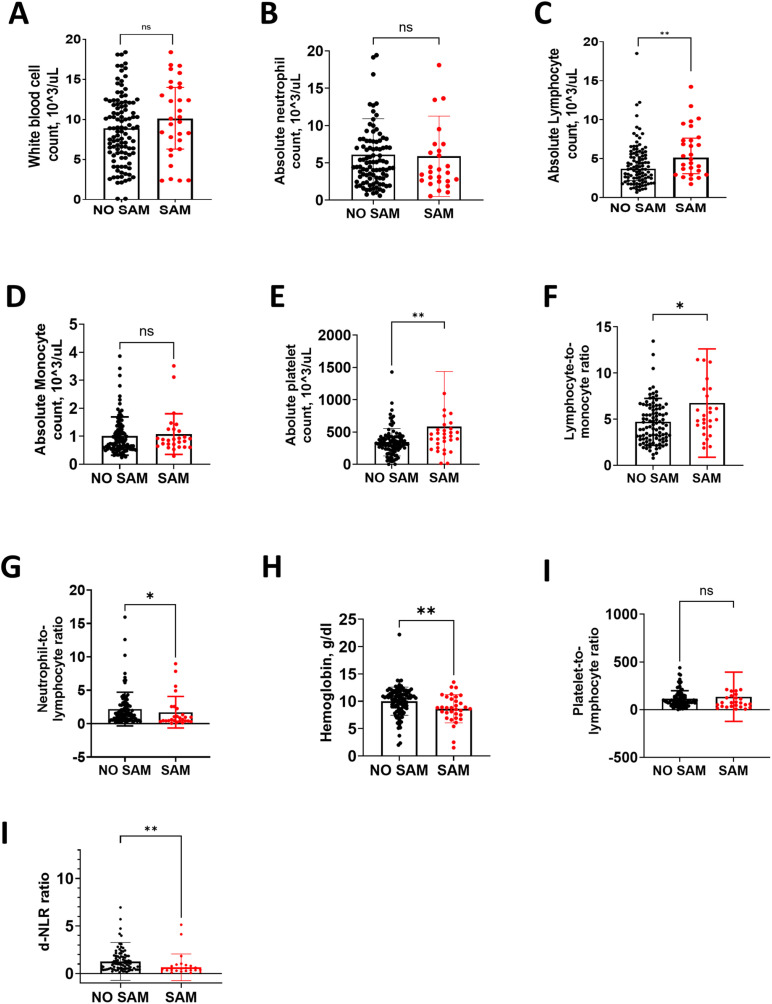
Laboratory characteristics between males with SAM and without SAM. This figure shows the median (interquartile range, IQR) between males without and with SAM, respectively: A) WBC, 8.9 (5.8, 12.1) vs. 10.1 (6.3, 14) x 10^3^/Ul, p = 0.185. B). ANC, 5.03 (2.9, 7.94) vs. 3.76 (2.53, 7.51) x 10^3^/Ul, p = 0.466. C). ALC, 3.68 (2.19, 5.58) vs. 5.13 (3.19, 7.53) x10^3^/Ul, p = 0.006. D). AMC, 0.78 (0.54, 1.23) vs. 0.88 (0.64, 1.25) x103/Ul, p = 0.331. E). APC, 309 (235, 424.5) vs. 436 (255, 551) x10^3^/Ul, p = 0.006**. F).** LMR, 4.33 (2.84, (6.2) vs. 4.96 (4.05, 8.26, p = 0.050. G). NLR, 1.38 (0.63, 2.74) vs. 0.61(0.40, 2.25, p = 0.022. H). HbG, 10.5 (8.7,11.6) vs. 8.8 (7.5,10.4) g/dl, p = 0.003. I). PLR, 90.8 (58.9, 144.9) vs. 71.2 (33.7, 143.8), p = 0.201. J). d-NLR, 0.91 (0.40, 1,80) vs. 0.38 (0.27, 0.78), p = 0.007. WBC, white blood cell; ALC, absolute lymphocyte; AMC, absolute monocyte count; APC, absolute platelet count; LMR, lymphocyte-monocyte ratio; NLR, neutrophil-lymphocyte ratio; HBG, hemoglobin; PLR, platelet-lymphocyte ratio; d-NLR, derived neutrophil-lymphocyte ratio. *p < 0.05, **p < 0.01, **p < 0.001.

**Fig 3:**
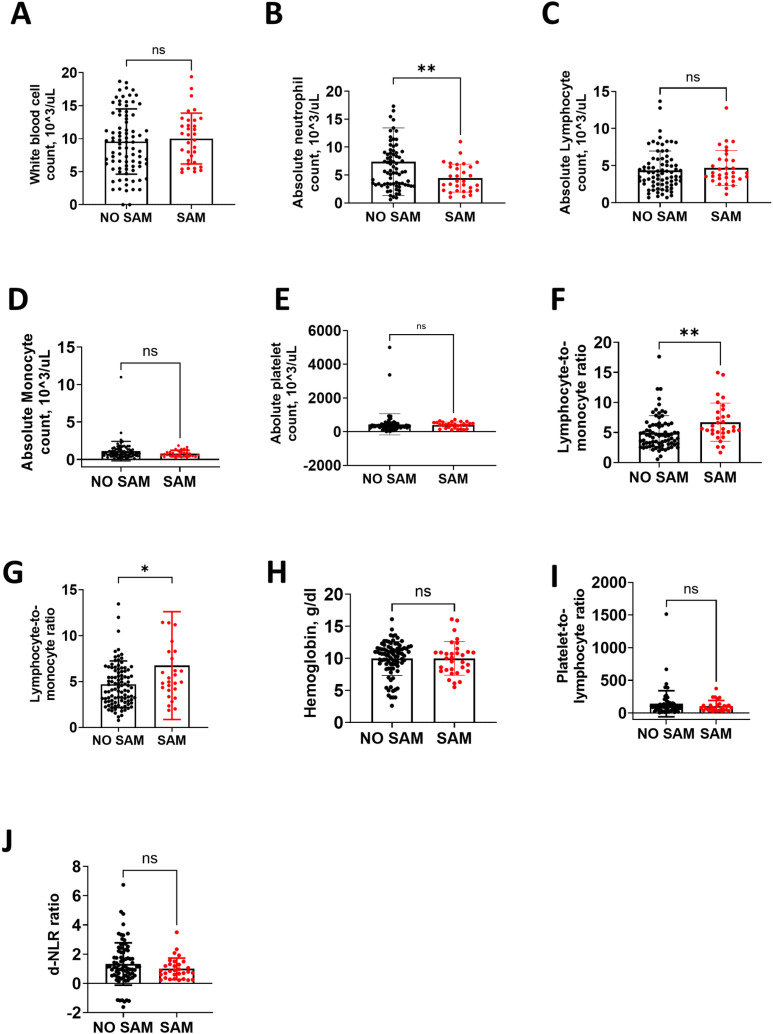
Laboratory characteristics between females with SAM and without SAM. This figure shows the median (interquartile range, IQR) between females without and with SAM, respectively: A) WBC, 9.5 (5.85, 13.6) vs. 10.2 (6.4, 12.8) x 103/Ul, p = 0.554. B). ANC, 5.72 (3.35, 9.2) vs. 3.89 (2.26, 6.48) x 103/Ul, p = 0.006. C). ALC, 3.95 (2.53, 5.55) vs. 4.01 (3.26, 5.75) x103/Ul, p = 0.366. D). AMC, 0.83 (0.52, 1.34) vs. 0.63 (0.46, 0.98) x10^3^/Ul, p = 0.182. E). APC, 317 (239.5, 427) vs. 418 (222, 540) x103/Ul, p = 0.155. F). LMR, 4.44 (3.05, 6.27) vs. 5.62 (4.92, 8.39), p = 0.001. G). NLR, 1.55 (0.97, 3.08) vs. 0.94 (0.41, 1.55), p = 0.003. H). HBG, 10.7 (9.1, 11.6) vs. 10.0 (8.1, 10.95) g/dl, p = 0.411. I). PLR, 98.9 (51.8, 135.4) vs. 81.8 (47.54, 119.4), p = 0.519. J). d-NLR, 1.13 (0.53, 2.11) vs. 0.83 (0.36, 1.30), p = 0.152. WBC, white blood cell; ALC, absolute lymphocyte; AMC, absolute monocyte count; APC, absolute platelet count; LMR, lymphocyte-monocyte ratio; NLR, neutrophil-lymphocyte ratio; HBG, hemoglobin; PLR, platelet-lymphocyte ratio; d-NLR, derived neutrophil-lymphocyte ratio. *p < 0.05, **p < 0.01, **p < 0.001.

Among males, high absolute platelet count (APC), high absolute lymphocyte count (ALC), low derived neutrophil-to-lymphocyte ratio (d-NLR), and low hemoglobin levels were associated with SAM when compared to those without SAM (Fig 2), but among females there were no significant differences in these hematological markers (Fig 3). Additionally, among females, low absolute-neutrophil-count (ANC) and high lymphocyte-to-monocyte ratio (LMR) were associated with SAM (Fig 3) but there was no significant difference in males (Fig 2). Low neutrophil-to-lymphocyte ratio (NLR) was associated with SAM in males and females when compared to males and females without SAM (Figs 2 and 3).

### Factors associated with SAM in males and females using logistic regression

On univariable analysis, HIV, TB, comorbidities, and high LMR were significantly associated with SAM in both males and females. Additionally, High APC, ALC, and low hemoglobin levels were only associated with SAM in males and not females. Among females, low ANC and NLR levels were significantly associated with SAM but not for males (Table 2).

**Table 2:** Univariable and multivariable logistic regression.

	Males				Females			
Variables	OR (95% CI)	P-value	AOR (CI)	P-value	OR (95% CI)	P-value	AOR (CI)	P-value
**Age, months**	0.98 (0.95, 1.00)	0.198	0.94 (0.89,1.00)	0.095	0.97 (0.94, 1.00)	0.095	0.91 (0.85, 0.97)	**0.009**
**HIV Status**								
Without HIV	Ref		Ref		Ref		Ref	
With HIV	10.3 (4.32,24.8)	**<0.001**	5.13 (0.91, 28.7)	0.062	3.05 (1.03, 8.96)	**0.048**	7.28 (0.99, 53.4)	0.050
**Tuberculosis**								
No	Ref		Ref		Ref		Ref	
Yes	13.1 (5.19, 33.0)	**<0.001**	14.3 (2.08, 98.5)	**0.006**	24.8 (6.81, 90.8)	**<0.001**	40.5 (4.83, 340)	**<0.001**
**Comorbidities**								
No	Ref		Ref		Ref		Ref	
Yes	2.10 (1.04, 4.23)	**0.037**	1.39 (0.36, 5.33)	0.627	3.60 (1.76, 7.38)	**<0.001**	4.1 (1.13, 14.9)	**0.031**
**APC, x10**^**3**^ **µl**	1.00 (1.00, 1.00)	**0.044**	1.00 (0.99, 1.00)	0.180	0.99 (0.99, 1.00)	0.696	–	–
**ANC, x10**^**3**^ **µl**	0.99 (0.90, 1.08)	0.990	–		0.83 (0.72, 0.95)	**0.010**	0.87 (0.76, 1.00)	0.050
**ALC, x10**^**3**^ **µl**	1.19 (1.03, 1.36)	**0.013**	0.98 (0.78, 1.23)	0.863	1.05 (0.89, 1.23)	0.526	–	–
**Hemoglobin,** *g/dl*	0.81 (0.69, 0.95)	**0.009**	0.85 (0.64, 1.14)	0.304	1.00 (0.85, 1.16)	0.978	–	–
**NLR**	0.90 (0.73, 1.12)	0.384	–	**–**	0.55 (0.36, 0.85)	**0.008**	1.23 (0.75, 2.01)	0.402
**LMR**	1.16 (1.01, 1.33)	**0.034**	1.39 (1.05, 1.83)	**0.017**	1.20 (1.04, 1.38)	**0.011**	1.22 (1.00, 1.49)	**0.045**
**d-NLR**	0.75 (0.52, 1.08)	0.128	–	**–**	0.80 (0.56, 1.14)	0.231	–	**–**

HIV, Human Immunodeficiency Virus, comorbidities (sickle cell disease, anemia, pneumonia, acute diarrhea disease, cerebral palsy, acute kidney injury, congenital heart disease, asthma, diabetes mellitus and lymphoma), WBC, white blood cell; ALC, absolute lymphocyte; ANC, absolute neutrophil count; APC, absolute platelet count; LMR, lymphocyte-monocyte ratio; NLR, neutrophil-lymphocyte ratio; PLR, platelet-lymphocyte ratio; d-NLR, derived neutrophil-lymphocyte ratio.

However, the multivariable analysis revealed that females with TB were 40 times more likely to develop SAM compared to females without TB, and males with TB had 14 times the odds of developing SAM in comparison to males without TB. Females with comorbidities were 4 times more likely to develop SAM than females without comorbidities, but males had no association with comorbidities. High LMR levels remained significantly associated with SAM in both males and females. Also, with increased age the odds of SAM developing in females were reduced by 9%, but age showed no association in males (Table 2).

## Discussion

This study highlights significant sex differences in SAM’s prevalence and risk factors in children under five years of age at Livingstone University Teaching Hospital.

Females were more frequently diagnosed with SAM in comparison to males, and this finding was contrary to most studies that found a prevalence higher in males than females [7,11,15]. These findings could be attributed to the fact that this population of females had a higher prevalence of comorbidities in comparison to males. This further implied that females are more likely to face severe complications when with SAM. However, this was contrary to some studies which found that males are generally more vulnerable to undernutrition and infectious diseases in comparison to females due to many factors such as genetic influence, as well as immune and endocrine factors. By virtue of females having two X-chromosomes, they are known to be genetically predisposed to mounting stronger immune responses to infections than males, aside from the influence of sex hormones like estrogen. Although sex hormones like estrogen and testosterone are low in young children, the levels are enough to influence the immune system [7,16]. In addition to biological factors, females are at risk due to social and cultural influences that play a role [9]. Patriarchal norms often prioritize male consumption of food, relegating female members to eat only after the males have eaten [9]. Traditional taboos also restrict women from consuming certain foods [9,17]. Furthermore, despite women comprising over half of the agricultural workforce, they have limited control over resource allocation, which affects their access to nutritious food [9]. Additionally, females have limited decision-making power, particularly in households, can delay their access to healthcare, leading to slower treatment for malnutrition-related conditions [18].

The association of TB with SAM was notable in both males and females, and it is known that the relationship between TB and SAM is complex and bidirectional, as each condition tends to exacerbate the others severity and progression leading to a poor outcome [13,19,20]. Malnourished individuals tend to have low leptin levels and reduced energy, which impairs macrophage activation, T-cell proliferation, and mechanistic target of rapamycin (mTOR) signalling [21,22]. Additionally, zinc deficiency is crucial as it affects the function of NADPH oxidase, which produces reactive oxygen species (ROS) necessary for the killing of Mycobacterium TB [23,24]. On the other hand, TB leads to malnutrition by triggering the release of pro-inflammatory cytokines such as TNF-α (tumor necrosis factor-alpha), IL-6, and IFN-γ, which increase basal metabolic rate, leading to excessive energy consumption. These cytokines also promote lipolysis and muscle protein degradation, resulting in cachexia [25,26]. In our setting, where TB is highly prevalent, screening for TB cases should be intensified, as most cases present without systemic signs and symptoms. This can be achieved through integrated healthcare models for TB and SAM in order to prevent adverse clinical outcomes [19].

HIV is known to be associated with SAM and though this association has been reported by many studies, sex differences have not been thoroughly explored [14,27,28]. HIV was associated with SAM in univariable analysis for both males and females and despite a higher prevalence displayed in males, there was no association in multivariable analysis. This implied that while HIV contributes to malnutrition, its impact may be moderated by other, more influential conditions or factors.

Increased age in females was associated with reduced odds of SAM in comparison to males in which age was of no significance. This implied that young age in females predisposes one to SAM. But currently, there is limited information in terms of age and sex differences, highlighting the need for further research in this area.

Males with SAM exhibited lower hemoglobin levels in comparison to females with SAM, this was in line with some studies that also found low hemoglobin levels in males [12,29]. Anemia is a known common comorbidity in children with SAM and it is known to predispose malnourished children to morbidity and mortality [30,31]. Hence, this finding implied that anemia may contribute to SAM in males and that there might be a need for early interventions to improve hematological outcomes in males with SAM, as anemia is known to complicate recovery, thus there is need to prevent and treat anemia in SAM children, including nutritional supplementation, fortification programs, and improved maternal nutrition [32]. On univariable analysis, high levels of hemoglobin demonstrated lower odds of having SAM in males. However, on multivariable analysis, there was no significance.

Lymphocyte-to-monocyte ratio is a known immune marker that assesses immune function and inflammation and serves an indicator of nutritional status and provides insights into disease prognosis and severity [33,34] In this study, a high LMR was associated with SAM in both males and females, suggesting that immune system dysfunction plays a key role in its progression by indicating an imbalance in the immune response. Similarly, a study by Wu et al. (2022) on participants with cirrhosis found that a high LMR was associated with an increased risk of malnutrition. However, there are limited studies examining the relationship between SAM and the monocyte-to-lymphocyte ratio (MLR) and among different sex [34]. Furthermore, on univariable analysis, high APC and ALC were associated with SAM in males, and for females, an increase in ANC and NLR reduced the odds of SAM. However, these associations lost significance in multivariable analysis. These markers have not yet been established in SAM. This study provides a foundation on which further research can be conducted to establish markers for SAM and thereby improve the clinical management of these patients in our setting which remains a challenge.

### Clinical implications

Sex-specific protocols that recognize risk profiles should be implemented to enable healthcare providers to proactively address SAM in males and females. The higher prevalence of SAM in females indicates the need for regular nutritional screening in females during early childhood, as this is the period when energy and nutrient demands are elevated. Additionally, early screening in females should prioritize those with comorbidities, more particularly TB as well as those of young age.

Given that TB is a critical risk factor for both males and females. Treatment protocols addressing both malnutrition and infection should be integrated. Aside from early detection and control of TB, treatment of TB should not only be pharmacologically based but should also include nutritional support to help restore immune function and improve overall health outcomes.

In resource-limited settings, immune markers such as LMR can be incorporated into standard routine assessments and possibly serve as an early adjunct marker for clinicians to assess the risk of malnutrition or, possibly serve as a measure of response to treatment in both males and females, especially in those with comorbidities.

### Study strengths and limitations

This study fills an important knowledge gap in understanding and identifying differential risk factors for SAM in males and females, as it is the first of its kind pioneering focus on specific sex differences in SAM in this region as well as specific to the catchment area of LUTH. Additionally, this approach provides a foundation for sex-specific tailored treatment and prevention strategies that uniquely address the needs of males and females affected by SAM in this region where SAM is known to be prevalent.

Currently, there is limited data on the relationship between SAM and these immune markers, highlighting the need for further research in this area, as these results indicate that hematological markers may also play a significant role in differentiating SAM between sexes.

Furthermore, this study also adds a unique dimension to understanding the relationship between immune function and SAM, highlighting the role of immune dysfunction in malnutrition and possibly offering potential clinical markers for early risk assessment and intervention, as it included an examination of key clinical and immune markers such as LMR, ALC, PLR, NLR, d-NLR, APC and AMC.

However, this study’s findings are specific to the catchment area of LUTH, which may limit the generalizability of the results to other regions. This population may have unique socio-demographic, cultural and healthcare access characteristics that do not represent those of children in other parts of Zambia or Sub-Saharan Africa.

This study provided valuable insights but it involved a relatively small sample size which limits the statistical power and may affect the reliability of the results. Hence, larger studies are needed to confirm these findings and provide more robust data on sex-specific risk factors in SAM.

As a retrospective, cross-sectional study, this study was limited to data from a single point in time, and its reliance on medical records posed challenges regarding accuracy and completeness. Hence, there is a need for a longitudinal approach to allow for the investigation of causality between risk factors and SAM as well as mitigate the limitation of missing or inaccurate information.

This study has a limited range of variables, it restricts a comprehensive understanding of all potential contributing factors and may overlook some underlying determinants of SAM that could differ by sex such as parental education, breastfeeding status, food security, and socioeconomic status. Furthermore, no correction for multiple comparisons, such as the Bonferroni adjustment, was applied in this analysis, which increases the risk of Type I errors. This should be considered when interpreting the findings.

## Conclusions

This study provides evidence of sex differences in the prevalence and risk factors of SAM in children under five years of age. Females are frequently diagnosed with SAM, most likely due to the presence of comorbidities such as TB and HIV. TB was found to be a critical risk factor for SAM in both males and females. These results implied that females with SAM are more likely to face additional complications and, further highlighted the need for sex-specific interventions in the management of SAM. For females, this includes targeted nutritional screening, provision of food hampers for those found at risk, and early linkage to healthcare facilities, as well as strengthening nutrition services for those who are HIV and TB positive.

## Supporting information

S1 Checklist STROBE checklist. (DOCX)

S1 Data Minimal dataset. (XLSX)
